# A Randomized, Double-Blind Pilot Study of Dose Comparison of Ramosetron to Prevent Chemotherapy-Induced Nausea and Vomiting

**DOI:** 10.1155/2015/523601

**Published:** 2015-09-03

**Authors:** Ka-Rham Kim, Gaeun Kang, Myung-Seo Ki, Hyun-Jeong Shim, Jun-Eul Hwang, Woo-Kyun Bae, Ik-Joo Chung, Jong-Keun Kim, Seongwook Jeong, Sang-Hee Cho

**Affiliations:** ^1^Department of Hemato-Oncology, Chonnam National University Medical School, Gwangju 519-763, Republic of Korea; ^2^Division of Clinical Pharmacology, Chonnam National University Hospital, Gwangju 519-763, Republic of Korea; ^3^Department of Pharmacology, Chonnam National University Medical School, Gwangju 519-763, Republic of Korea; ^4^Department of Anesthesiology and Pain Medicine, Chonnam National University Medical School, Gwangju 519-763, Republic of Korea

## Abstract

*Purpose*. This study was conducted to determine the optimal dose titration of ramosetron to prevent the Rhodes Index of Nausea, Vomiting, and Retching (RINVR). *Methods*. Patients treated with folic acid, 5-fluorouracil, and oxaliplatin were randomized into three groups (0.3 mg, 0.45 mg, and 0.6 mg ramosetron before chemotherapy). The pharmacokinetics and pharmacodynamics using RINVR were evaluated. *Results*. Seventeen, 15, and 18 patients received ramosetron at doses of 0.3 mg, 0.45 mg, and 0.6 mg, respectively. *T*_max_ (h), *C*_max_ (ng/mL), and AUC_last_ (ng·h/mL) were associated with dose escalation significantly, showing a reverse correlation with the RINVR during chemotherapy. Acute CINV was observed in four patients (22.2%), two patients (14.3%), and one (5.6%) patient and a delayed CINV on day 7 was found in eight (47%), three (21.4%), and five (27.8%) patients in each group. The complete response rate was increased with dose escalation (35.3%, 50.0%, and 72.2% in each group) and also showed the tendency for decreasing moderate-to-severe CINV. *Conclusions*. This study shows a trend regarding the dose-response relationship for ramosetron to prevent CINV, including delayed emesis. It suggested that dose escalation should be considered in patients with CINV in a subsequent cycle of chemotherapy, and an individual approach using RINVR could be useful to monitor CINV.

## 1. Introduction

Chemotherapy-induced nausea and vomiting (CINV) remains one of the most feared adverse events of chemotherapy among cancer patients [1]. Not only early onset CINV (acute, within 24 h after chemotherapy) but also delayed CINV (≥2 days after chemotherapy) can interfere with compliance of treatment and cause patients to receive delayed chemotherapy, contemplate refusing further treatment, and develop anticipatory CINV [2]. CINV is an autonomic reflex controlled by multiple neurotransmitter systems. Among them, the serotonin/5-hydroxytrptamine 3 receptor (5-HT_3_) and substance P/neurokinin-1 receptor (NK-1) systems have played important roles in controlling CINV, and blocking both systems has been demonstrated to reduce CINV in patients receiving chemotherapy [3–5]. Based on the guidelines of the National Comprehensive Cancer Network, patients receiving a moderately emetogenic chemotherapy (MEC) regimen are recommended a 5-HT_3_ receptor antagonist and dexamethasone, while those receiving a highly emetogenic chemotherapy (HEC) regimen are given a 5-HT_3_ receptor antagonist, a corticosteroid, and an NK-1 receptor antagonist [6]. As shown above, the 5-HT_3_ receptor antagonist is the most crucial drug to control CINV in a MEC or HEC regimen. However, even if the standard dose of the 5-HT_3_ receptor antagonist has been used, 20–30% of patients complain of nausea or vomiting in practice [7, 8], and studies concerning the optimal concentration of 5-HT_3_ receptor antagonists are lacking.

Ramosetron, a new member in the class of selective 5-HT_3_ receptor antagonists and is a tetrahydrobenzimidazole derivative structurally independent of the previously developed 5-HT_3_ receptor antagonists, such as ondansetron, granisetron, and tropisetron. Ramosetron is more potent and has longer-lasting effects than older agents because of a slower rate of dissociation from the target receptor and higher binding affinity [9]. 5-HT_3_ antagonists such as granisetron or palonosetron have been investigated extensively regarding the optimal dose to treat postoperative nausea and vomiting (PONV) or CINV. Generally, the dose required to prevent CINV is two- to threefold higher than that needed for PONV [10–12]. However, a similar dose of ramosetron has been used for both PONV and CINV, and no study has evaluated the optimal dose titration for ramosetron to prevent CINV.

To evaluate the efficacy of antiemetics, complete response (CR: no emesis and no rescue medication) and complete protection (CP: no emesis, no use of rescue medication, and no significant nausea) have been commonly used as meaningful indices [7, 13, 14]; nevertheless, they do not quantify mild-to-severe CINV. The Rhodes Index of Nausea, Vomiting, and Retching (RINVR) was developed by Rhodes and McDaniel to assess nausea or vomiting after surgery or the administration of chemotherapy [15]. In this scale, eight items to evaluate the experience of nausea/vomiting, occurrence of nausea/vomiting, and issues with nausea/vomiting are listed. A higher point indicates more intense CINV; therefore, the RINVR is used to assess the severity of CINV more precisely. In addition, antiemetics have been widely used; the incidence of vomiting has been decreased. In the contrary, nausea and retching have been major concerns and a new parameter for monitoring CINV is needed.

We hypothesized that if dose escalation of a 5-HT_3_ antagonist could be effective against CINV, the frequency or severity of CINV could also be reduced by subsequent dose increases in the next cycle of chemotherapy in patients who experienced moderate-to-severe CINV in the previous cycle. Based on this concept, the purpose of the present study was to evaluate the optimal concentration of ramosetron to prevent CINV using the RINVR clinical parameter based on pharmacokinetic (PK) and pharmacodynamic (PD) studies as a pilot trial.

## 2. Materials and Methods

### 2.1. Patients

To evaluate the efficacy of ramosetron for each dose, we limited the enrolled patients only to those receiving FOLFOX (folic acid, 5-fluorouracil, and oxaliplatin), which does not require inclusion of an NK-1 receptor antagonist during the first cycle. Patients were eligible if they were diagnosed with colon cancer and treated with FOLFOX as adjuvant chemotherapy after curative resection or palliation. Additionally, patients who were at least 19 years old and had an Eastern Cooperative Oncology Group performance score of 0–2 with adequate bone marrow and organ function were included in the present study (i.e., absolute neutrophil count ≥1,500/*μ*L, platelet count ≥100,000/*μ*L, serum bilirubin level <2.0 mg/dL, creatinine level <1.5 mg/dL, and serum transaminase levels less than twice the upper limit of normal). Patients were excluded from the study if they had obstructive symptoms due to previous stomach disease or intestinal adhesion, received radiotherapy, or sustained vomiting associated with abnormalities in the central nervous system. Other exclusion criteria included a history of another malignancy, pregnancy, or lactation, a history of or current distant metastasis, a history of clinically significant cardiac disease within the last 6 months, active serious infection, or a psychiatric illness that would preclude obtaining informed consent.

### 2.2. Ethics Statement

The current study was a single center, prospective, double-blind, randomized trial conducted at Chonnam National University Hwasun Hospital and the protocol was approved by the Institutional Review Board Committee (CNUHH-2012-103). The trial was registered in ClinicalTrial.gov (NCT02076529, URL: https://clinicaltrials.gov/ct2/show/NCT02076529?term=ramosetron&rank=1) and it was conducted in compliance with the good clinical practice guidelines issued by the International Conference on Harmonization of Technical Requirements for Registration of Pharmaceuticals for Human Use and the ethical principles of the Declaration of Helsinki. Written informed consent was obtained from all patients before the start of any screening procedure.

### 2.3. Study Design and Treatment

After obtaining informed consent from the patients, they were assigned randomly to receive a single intravenous (IV) dose of 0.3 mg, 0.45 mg, or 0.6 mg ramosetron administered 30 min before the first dose of chemotherapy on day 1. Dexamethasone 10 mg was also administered intravenously within 30 min before ramosetron injection to prevent CINV and anaphylaxis from oxaliplatin. The randomization scheme was determined by the study's biostatistician based on a permuted block design (*K* = 6). The treatment allocation scheme was maintained by the study pharmacist who assumed the responsibility for blinded drug distribution. After antiemetic preparation, all of the patients received FOLFOX4 chemotherapy consisting of leucovorin, 200 mg/m^2^/day given as a 2 h infusion, followed by a bolus of 5-fluorouracil, 400 mg/m^2^, and a 22 h continuous infusion of 600 mg/m^2^ 5-fluorouracil, repeated for two consecutive days. Oxaliplatin, 85 mg/m^2^, was administered on day 1 only as a 2 h infusion in 250 mL dextrose 5%, concurrently with leucovorin. Rescue antiemetics were permitted during treatment, and the results were recorded. All of the patients were required to be hospitalized for 2 days to assess RINVR and collect blood samples. In addition, follow-up examination was performed on day 8 (range, 6–10 days) to monitor the safety.

### 2.4. Blood Sample Preparation and Quantification of Ramosetron

Blood samples for PK analysis were drawn at 10 min, 1 h, 6 h, 24 h, and 48 h after the injection of ramosetron. The blood samples were collected into EDTA tubes. Each sample was then centrifuged at 1500 g for 10 min at 4°C. At least 0.4 mL plasma was harvested and stored at −70°C until analysis. The plasma concentrations of ramosetron were analyzed using validated high-performance liquid chromatography-tandem mass spectrometry (HPLC-MS/MS). Briefly, 200 *μ*L plasma sample was mixed with 20 *μ*L of an internal standard (5 ng/mL risperidone). Next, 1.5 mL methyl tert-butyl ether was added, and the mixture was vortexed for 2 min and centrifuged at 12,000 rpm for 2 min. The supernatants were transferred and evaporated under nitrogen. The residue was reconstituted in 100 *μ*L 40% acetonitrile, and 10 *μ*L of the latter mixture was injected into the HPLC-MS/MS system (HPLC system, Shimadzu LC-20A HPLC system (Shimadzu Co., Kyoto, Japan); MS/MS system, API 4000Q-TRAP mass spectrometer (AB SCIEX, Framingham, MA, USA)). The column was a Gemini C18 2.0 × 100 mm (Phenomenex, Torrance, CA, USA), and the mobile phase consisted of ammonium acetate (10 mM) and acetonitrile (40 : 60, v/v). The flow rate was maintained at 0.25 mL/minute. Linear calibration curves were validated in the ranges of 0.1–100 ng/mL for ramosetron (*r* ^2^ = 0.98). The intra- and interday precision and accuracy were verified within 15% and 85–115%, respectively.

### 2.5. Pharmacokinetic Analysis

The pharmacokinetic parameters of ramosetron were analyzed by noncompartmental methods using WinNonlin version 6.3 (Pharsight Co., Mountain View, CA, USA). The maximum concentration (*C*_max_) and time to *C*_max_ (*T*_max_) were obtained directly from the concentration-time data. The area under the concentration-time curve of ramosetron from zero to the last measurable concentration (AUC_last_) was calculated using the linear trapezoidal rule. From the terminal slope of the concentration time curve, the elimination rate constant was estimated using linear regression, and the terminal half-life (*T*_1/2_), clearance (CL), and volume of distribution at the steady state (*V*_ss_) were calculated.

### 2.6. Evaluation of Nausea/Vomiting and Safety

The RINVR, used to assess the degree of CINV, comprises eight questions, each with a total of five choices rating the frequency and intensity of symptoms. Among the survey questions, numbers 1, 4, 6, 7, and 8 address the subclass of symptom occurrence, and numbers 2, 3, and 5 address the subclass of symptom distress by assessing the degree of discomfort caused by the symptoms. The scores from all eight questions are then summed to obtain the subclass of symptom experience points. Therefore, the total sum of the nausea, vomiting, and retching experience points is defined as RINVR point. RINVR was recorded during an interview at 1 h, 6 h, 24 h, and 48 h after starting chemotherapy. Seven days after starting chemotherapy, the RINVR was reassessed by telephone interview to evaluate the presence of delayed emesis. The RINVR score was divided into four groups for comprehensive analysis as follows: none (0), mild (1–8), moderate (9–16), and severe (17–32). A CR was defined as no emetic episode and no use of rescue medication during the first cycle of chemotherapy.

Safety was evaluated based on the results of laboratory tests and electrocardiography. The signs and symptoms were graded according to the Common Terminology Criteria for Adverse Events (CTCAE), version 4.0.

### 2.7. Statistics

This study was planned as a pilot study to evaluate the efficacy of dose escalation of ramosetron. Since there is no data on target population to use for sample size calculation for pilot study, we adopt sample size that was used in a previous similar granisetron study [16]. Data are presented as mean ± SD for continuous data and absolute frequencies (*n*) and percentages for frequency data. To assess the pharmacokinetic linearity, statistical comparisons of the dose-normalized *C*_max_ (*C*_max_/*D*), dose-normalized AUC (AUC_last_/*D*),* T*_1/2_, CL, and *V*_ss_ among the ramosetron dose groups were performed using the Kruskal-Wallis test. One-way analysis of variance (with Bonferroni post hoc test) was utilized to assess the differences in patient characteristics and to compare the RINVR score and concentration of ramosetron. *χ* ^2^ test or Fisher's exact probability test was used to compare the proportions of categorical variables. The number of emetic episodes, severity of nausea, and RINVR were compared among the ramosetron dose groups using the Kruskal-Wallis test. Statistical significance was recognized when the *P* value was less than 0.05. SPSS version 21.0 (IBM, Inc., Chicago, IL, USA) was utilized for statistical analyses.

## 3. Results

### 3.1. Patient Characteristics

From October 2012 to October 2013, fifty-one patients were enrolled, and one patient refused to participate in the present study; therefore, the data from 50 patients were analyzed. Seventeen, 15, and 18 patients received ramosetron at a dose of 0.3 mg, 0.45 mg, and 0.6 mg, respectively. Their baseline clinical characteristics are summarized in Table 1. There was no difference between the groups in terms of sex, age, weight, height, or body surface area (BSA). Although female is well known risk factor for CINV, thirty-three patients (66%) were male in present study. This result is associated with colon cancer prevalence which is more common in male than female. Thirty-eight patients (74%) were diagnosed with stage III colon cancer after curative resection, and 12 patients (26%) with stage IV disease. Patients with nausea, vomiting, or retching despite using ramosetron received metoclopramide, domperidone maleate, or lorazepam as rescue medication. There was no significant difference in the frequency of the rescue dose among the ramosetron dose groups.

### 3.2. Pharmacokinetic Analysis

**Figure 1:**
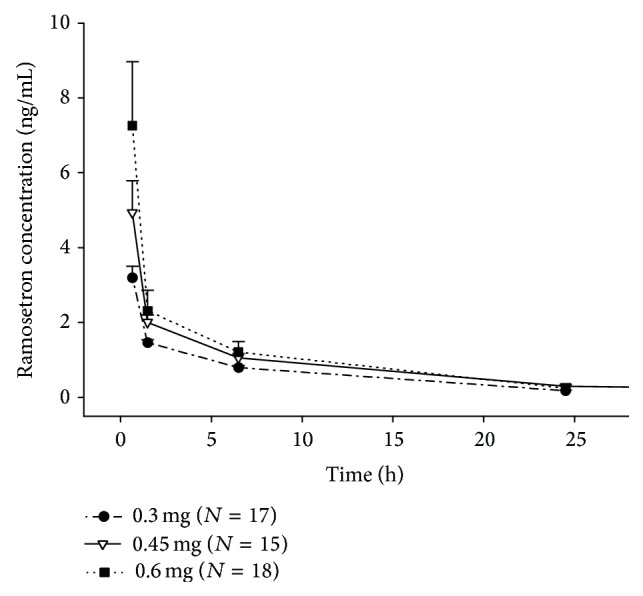
Mean plasma concentration-time profiles of ramosetron after a single intravenous injection of 0.3, 0.45, and 0.6 mg of ramosetron. Bars represent standard errors.

The mean plasma concentration-time profiles of ramosetron at doses of 0.3 mg, 0.45 mg, and 0.6 mg are shown in Figure 1.  Table 2 shows the arithmetic mean ± standard deviations (SDs) of the pharmacokinetic parameters determined using noncompartmental analysis. A dose-dependent increase in the mean *C*_max_ and AUC_last_ of ramosetron is shown in all three dose groups. The mean *C*_max_ ranged from 3.2 to 7.3 ng/mL, and the mean AUC_last_ ranged from 17.9 to 35.2 ng·h/mL. The mean clearance (CL) and volume of distribution at the steady state (*V*_ss_) were in the ranges of 17.1–22.5 L/h and 110.6–142.3 L/h, respectively, for the three ramosetron dose groups. There were no significant differences in the *C*_max_/*D* (*P* = 0.450), AUC_last_/*D* (*P* = 0.301),* T*_1/2_ (*P* = 0.845), CL (*P* = 0.235), or *V*_ss_ (*P* = 0.361) among the ramosetron dose groups, indicating that ramosetron exhibited linear pharmacokinetic properties.

### 3.3. Efficacy of Ramosetron in Dose Escalation

**Figure 2:**
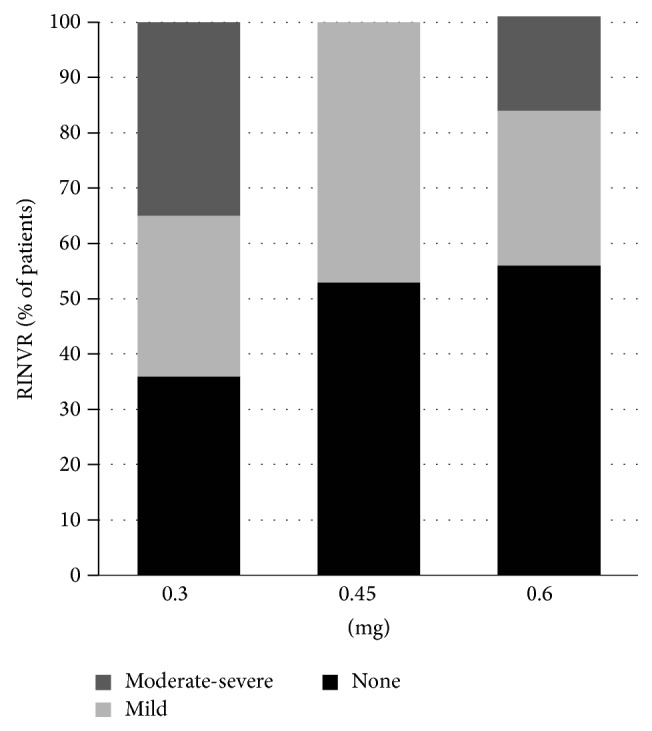
Total Rhodes Index of Nausea, Vomiting, and Retching (RINVR) score during 7 days according to ramosetron dose group (*n* = 50).

Nausea is the most common symptom of CINV (40%), followed by retching (18%) and vomiting (8%). None of the patients had CINV during the first 6 h after starting chemotherapy. CINV showed an increasing tendency for 7 days. The total RINVR scores at 1 h, 6 h, 24 h, 48 h, and 7 days after starting chemotherapy are shown in Table 3. There was no statistical significance between the RINVR and dose escalation, but a reverse correlation with RINVR was found at 1 day (*P* = 0.237) and 7 days (*P* = 0.377) after starting chemotherapy according to dose escalation. Twenty-five patients (80%) had CINV of at least one point on the RINVR, among whom nine patients (18%) showed moderate or severe RINVR. One day after starting chemotherapy, four patients (22.2%), two patients (14.3%), and one (5.6%) patient who received 0.3 mg, 0.45 mg, and 0.6 mg of ramosetron, respectively, showed CINV. Delayed CINV 7 days after chemotherapy occurred in eight (47%), three (21.4%), and five (27.8%) patients in the respective ramosetron groups. Patients who received a higher dose of ramosetron showed a greater trend for a CR than did patients who received a lower dose of ramosetron (Figure 2). Regarding the severity, all symptoms for 2 days were graded as mild RINVR. However, five (29%) and three patients (17%) in the 0.3 and 0.6 mg ramosetron-treated groups, respectively, showed moderate RINVR on day 7. Severe RINVR was seen in only one patient who was treated with 0.3 mg ramosetron.

### 3.4. Safety

Of the 50 patients, there were no grade 3 or 4 adverse events. Constipation and elevated liver enzymes were common symptoms, but most of these were considered to be unrelated to ramosetron and instead were associated with underlying disease or chemotherapy. One patient showed QT prolongation on electrocardiography; however, he had no subjective symptoms, and the condition disappeared on the next cycle of chemotherapy. The incidence and grade of events were similar among the three dose groups (Table 4).

## 4. Discussion

When the role of chemotherapy is expanded as an adjuvant or for palliation, the willingness to preserve the quality of life and the completion of planned chemotherapy are also increased. Although 5-HT_3_ antagonists and neurokinin-1 inhibitors can provide better supportive care than they did previously [7, 17], limitations of these drugs persist. CINV is associated not only with the chemotherapeutic regimen but also with patient characteristics, such as previous morning sickness, age, gender, performance status score, and smoking or alcohol habits [18]. The standard dose of antiemetics would not be sufficient for high-risk patients, and the precision marker to predict these high-risk patients has not been identified. Therefore, the meticulous and individual approach used for patients with CINV in the previous cycle would be important to prevent further CINV on subsequent cycles.

Several studies concerning the dose escalation of antiemetics to prevent CINV have been performed [19, 20]. The first generation of 5-HT_3_ antagonists, such as ondansetron, dolasetron, granisetron, and tropisetron, has been very effective in the control of acute emesis but has not been as effective against delayed emesis [21, 22]. Among 5-HT_3_ antagonists, ramosetron and palonosetron are recently developed agents. Ramosetron has been shown to have higher selectivity for serotonin than other first generation 5-HT_3_ antagonists showing superior efficacy in the acute and delayed CINV than other first generation 5-HT_3_ antagonists. Palonosetron, which is second generation of 5-HT_3_ antagonists, has been investigated extensively in the setting of PONV and CINV as a recommended dose of 0.075 mg IV and 0.25 mg IV, respectively. Compared with palonosetron, the study of ramosetron has been limited, and the recommended dose of ramosetron is 0.3 mg IV in both PONV and CINV. In a previous study evaluating the efficacy of different doses of palonosetron (0.075 mg, 0.25 mg, and 0.75 mg IV in each group), CR was achieved in 44.4% of the 0.25 mg group and 59.3% of the 0.75 mg group in patients receiving Adriamycin/carboplatin or epirubicin/carboplatin. This was very similar to the current results showing a CR of 35.3% in the 0.3 mg group and 61.1% in the 0.6 mg group. Not only dose escalation but also the repeated administration of palonosetron improved CINV using 0.75 mg IV on days 1 and 3 [23]. As a result, CR occurred in 76.9% of patients who received MEC or HEC. The studies that used high doses of palonosetron up to sixfold the standard dose suggest that dose escalation of a 5-HT_3_ antagonist could improve CINV. In the present study, twice the standard dose (0.6 mg) of ramosetron resulted in better clinical outcomes compared with the standard dose (0.3 mg) in chemotherapy-naïve patients. This suggests that the dose of ramosetron needed to be escalated to control CINV or escalated individually in patients that experienced CINV, despite the prevention using a standard dose of ramosetron. Although the stratification was not performed according to gender, age, height, weight, or BSA, there was no significant difference in each dose group. In pharmacokinetic analyses, the current study showed the highest AUC_last_ of ramosetron in the 0.6 mg group among the three dosing groups; therefore, the 0.6 mg group had a higher exposure to ramosetron than did the low-dose group when the other conditions were consistent.

In addition to clinical effectiveness for CINV, the current study revealed that RINVR is a useful tool to monitor CINV. In addition to CR or CP, the National Cancer Institute-Common Terminology Criteria (NCI-CTC) have been used to monitor nausea or vomiting related to chemotherapy toxicities. However, it is not completely sufficient to reflect the CINV based only on its frequency and degree of nausea and vomiting or retching. The current study showed improvement in the RINVR according to the dose of ramosetron with moderate-to-severe RINVR in six (35%) and three patients (17%) treated with 0.3 mg and 0.6 mg of ramosetron on day 7, respectively. These results suggest that even though the complete inhibition of CINV was not achieved, a high dose of ramosetron could attenuate the degree of CINV; therefore, RINVR could be another valuable tool to assess CINV.

Based on the pattern of CINV, nausea is the most common symptom and vomiting the least prevalent symptom according to the RINVR scores. The cause might be related to the MEC regimen used in this study, and the routine use of antiemetics can prevent vomiting, but the prevalence of nausea and retching was increased relatively. In addition, the RINVR score showed an increasing tendency over 7 days, and 47% of the patients who received 0.3 mg ramosetron had CINV at least of one score of RINVR; in contrast, 28% of the patients who received 0.6 mg ramosetron experienced CINV on day 7. Although the concentration of ramosetron was cleared within the first 2 days according to the pharmacokinetic data, this result suggested that the control of acute emesis could affect the delayed emesis, and a high dose of ramosetron could help to reduce the delayed emesis. In consideration of the prevalence of delayed emesis, the study about the usefulness of dose escalation of ramosetron or introduction of NK-1 receptor antagonist would be helpful to prevent delayed emesis. As a limitation of this study, there was no statistically significant difference in CINV based on RINVR among the three dose groups. The reason for this might be related to the small number of patients in each group, MEC regimen which could be related to fewer CINV events compared with an HEC regimen, and small number of female patients who are more prone to CINV, thus diluting the efficacy of the antiemetics. Further large-scale studies are needed to confirm the efficacy of dose escalation in the subsequent chemotherapy for patients who showed moderate-to-severe CINV in previous chemotherapy.

## 5. Conclusion

This pilot study showed dose-dependent pharmacokinetics of ramosetron with a better RINVR trend according to the dose escalation with a good safety profile. In addition, RINVR would be a useful tool to assess the quantification of CINV. Based on this pilot trial, further large-scale study is needed for more robust clinical outcomes.

## Figures and Tables

**Table 1:** Clinical characteristics.

	0.3 mg	0.45 mg	0.6 mg	*P* value
	(*N* = 17)	(*N* = 15)	(*N* = 18)	
Male	8 (53.3%)	12 (80.0%)	13 (72.2%)	0.157
Age (year)	58.94 ± 9.74	61.64 ± 7.76	57.72 ± 10.13	0.547
Weight (kg)	63.82 ± 11.83	62.94 ± 10.34	61.82 ± 9.07	0.385
Height (cm)	162.72 ± 4.92	164.31 ± 7.67	163.62 ± 7.55	0.885
BSA (m^2^)	1.69 ± 0.18	1.70 ± 0.16	1.67 ± 0.18	0.923
Colon cancer				
Stage III	12 (71%)	12 (80%)	14 (78%)	0.895
Stage IV	5 (29%)	3 (20%)	4 (22%)	
Use of rescue antiemetic	3 (17.6%)	5 (33.3%)	3 (16.7%)	0.810

Data are presented as mean ± standard deviation.

BSA, body surface area

**Table 2:** Pharmacokinetic parameters of ramosetron after a single intravenous injection of 0.3, 0.45, and 0.6 mg of ramosetron.

	0.3 mg	0.45 mg	0.6 mg
	(*N* = 17)	(*N* = 15)	(*N* = 18)
*C*_max⁡_ (ng/mL)	3.2 ± 1.3	4.9 ± 3.3	7.3 ± 9.3
*T*_max⁡_ (h)	0.67	0.67	0.67
AUC_last_ (ng·h/mL)	17.9 ± 6.9	25.9 ± 16.0	35.2 ± 34.8
*T*_1/2_ (h)	6.4 ± 2.2	6.5 ± 3.2	6.4 ± 1.8
CL (L/h)	17.1 ± 6.6	21.3 ± 13.6	22.5 ± 11.8
*V*_ss_ (L)	110.6 ± 38.7	128.5 ± 60.6	142.3 ± 86.6

Data are presented as mean ± standard deviation.

*C*_max⁡_, maximum concentration; *C*_max⁡_/*D*, maximum concentration/dose; *T*_max⁡_, time to *C*_max⁡_; AUC_last_, area under the concentration-time curve of ramosetron from zero to the last measurable concentration; AUC_last_/*D*, area under the concentration-time curve of ramosetron from zero to the last measurable concentration/dose; *T*_1/2_, terminal half-life; CL, clearance; *V*_ss_, volume of distribution at steady state.

**Table 3:** Rhodes Index of Nausea, Vomiting, and Retching (RINVR) scores among 0.3 mg, 0.45 mg, and 0.6 mg dosing groups at 1 hour, 6 hours, 1 day, 2 days, and 7 days after starting chemotherapy.

	0.3 mg	0.45 mg	0.6 mg
	(*N* = 17)	(*N* = 15)	(*N* = 18)
1 hr	0.00 ± 0.00	0.00 ± 0.00	0.00 ± 0.00
6 hr	0.00 ± 0.00	0.00 ± 0.00	0.00 ± 0.00
1 day	0.78 ± 1.56	0.36 ± 0.93	0.11 ± 0.47
2 days	1.11 ± 2.52	0.79 ± 1.12	0.44 ± 1.34
7 days	3.61 ± 4.80	0.71 ± 1.44	2.39 ± 4.07

Data are presented as mean ± standard deviation.

**Table 4:** Treatment related adverse events (*n* = 50).

	Dose of ramosetron					
	0.3 mg, *n* (%)		0.45 mg, *n* (%)		0.6 mg, *n* (%)	
	Grade 1	Grade 2	Grade 1	Grade 2	Grade 1	Grade 2
AST	1 (6)	1 (6)	0	0	1 (6)	0
ALT	2 (12)	1 (6)	1 (7)	0	2 (11)	0
r-GTP	1 (6)	1 (6)	0	0	1 (6)	0
Constipation	3 (18)	1 (6)	0	0	3 (17)	0
Rash	0	0	1 (7)	0	0	0
ECG QT prolongation	0	0	0	0	1 (6)	0
